# Computer-extracted global radiomic features can predict the radiologists’ first impression about the abnormality of a screening mammogram

**DOI:** 10.1093/bjr/tqad025

**Published:** 2023-12-12

**Authors:** Somphone Siviengphanom, Sarah J Lewis, Patrick C Brennan, Ziba Gandomkar

**Affiliations:** Medical Image Optimisation and Perception Group, Discipline of Medical Imaging Science, Sydney School of Health Sciences, Faculty of Medicine and Health, The University of Sydney, Sydney, NSW 2006, Australia; Medical Image Optimisation and Perception Group, Discipline of Medical Imaging Science, Sydney School of Health Sciences, Faculty of Medicine and Health, The University of Sydney, Sydney, NSW 2006, Australia; Medical Image Optimisation and Perception Group, Discipline of Medical Imaging Science, Sydney School of Health Sciences, Faculty of Medicine and Health, The University of Sydney, Sydney, NSW 2006, Australia; Medical Image Optimisation and Perception Group, Discipline of Medical Imaging Science, Sydney School of Health Sciences, Faculty of Medicine and Health, The University of Sydney, Sydney, NSW 2006, Australia

**Keywords:** radiomics, screening mammograms, gist, breast cancer, machine learning, global radiomic features, current mammograms, prior mammograms

## Abstract

**Objective:**

Radiologists can detect the gist of abnormal based on their rapid initial impression on a mammogram (ie, global gist signal [GGS]). This study explores (1) whether global radiomic (ie, computer-extracted) features can predict the GGS; and if so, (ii) what features are the most important drivers of the signals.

**Methods:**

The GGS of cases in two extreme conditions was considered: when observers detect a very strong gist (*high-gist*) and when the gist of abnormal was not/poorly perceived (*low-gist*). Gist signals/scores from 13 observers reading 4191 craniocaudal mammograms were collected. As gist is a noisy signal, the gist scores from all observers were averaged and assigned to each image. The *high-gist* and *low-gist* categories contained all images in the fourth and first quartiles, respectively. One hundred thirty handcrafted global radiomic features (GRFs) per mammogram were extracted and utilized to construct eight separate machine learning random forest classifiers (*All*, *Normal*, *Cancer*, *Prior-1*, *Prior-2*, *Missed*, *Prior-Visible*, and *Prior-Invisible*) for characterizing *high-gist* from *low-gist* images. The models were trained and validated using the 10-fold cross-validation approach. The models’ performances were evaluated by the area under receiver operating characteristic curve (AUC). Important features for each model were identified through a scree test.

**Results:**

The *Prior-Visible* model achieved the highest AUC of 0.84 followed by the *Prior-Invisible* (0.83), *Normal* (0.82), *Prior-1* (0.81), *All* (0.79), *Prior-2* (0.77), *Missed* (0.75), and *Cancer* model (0.69). *Cluster shade*, *standard deviation*, *skewness*, *kurtosis*, and *range* were identified to be the most important features.

**Conclusions:**

Our findings suggest that GRFs can accurately classify *high-* from *low-gist* images.

**Advances in knowledge:**

Global mammographic radiomic features can accurately predict *high-* from *low-gist* images with five features identified to be valuable in describing *high-gist* images. These are critical in providing better understanding of the mammographic image characteristics that drive the strength of the GGSs which could be exploited to advance breast cancer (BC) screening and risk prediction, enabling early detection and treatment of BC thereby further reducing BC-related deaths.

## Introduction

Clinicians such as radiologists, the experts in the interpretation of medical images, often report that they have an initial impression about an abnormality of a medical image or its “gist” after momentarily glancing at an image. Several observer studies provided evidence suggesting that in less than a half-second, radiologists are capable of distinguishing abnormal mammograms^1^ or chest X-rays from the normal ones.^2^ A study focusing on mammographic images indicated that radiologists can distinguish abnormal from normal mammograms, containing no obvious localized sign of cancer, contralateral to the malignancies.^3^ It was also shown that based on a half-second image presentation, the gist of the abnormal can be detected in prior normal breast images belonging to women who were later diagnosed with breast cancer (BC) (ie, missed cancers based on prior images from a previous screening round).^4–6^ Moreover, previous studies showed that women with a false positive (FP) result on screening mammogram or who have had a cancer in one breast have elevated risk of developing a future/contralateral BC.^7^^,^^8^ These findings imply that the initial impression of a radiologist does not necessarily have a localized source within the image but is possibly driven by the mammographic textural features and not associated with other risk factors such as breast density.^9–12^

In medical image perception literatures, the holistic perceptual processing of radiological instantaneous view of an image’s abnormality features is often called the gist signal, which has been extensively studied to better understand radiological errors and enhance screening performance.^3–6^^,^^11-21^ From the human visual system, one may claim that gist contains both global and local source of information about “what” and “where” the abnormality in an image may be, correspondingly.^12^^,^^22^^,^^23^ A large body of gist literature, studying gist on images without a localized cancer sign such as contralateral mammograms, revealed that gist is associated with global “what” information occurring without prior location of that information.^3–5^^,^^13-15^^,^^23^^,^^24^ Views from the basic vision science domains suggest that gist information can be rapidly extracted (eg, using just as small as a half-second of an image viewing time) from the entire global image properties in a “non-selective” manner, while identification of the information occurs from the local properties (ie, the “selective” pathway).^16^^,^^17^^,^^25^^,^^26^ With that, gist is evidently a global rapid signal which will be referred to as the “global gist signal” (GGS) in this article.

Numerous gist studies focusing on the process of understanding natural scenes suggest that only after a brief sight of an image, observers are capable of categorizing these images into classes such as indoor or outdoor.^27^^,^^28^ The human visual system relies on texture features in recognizing the global gist of the natural scenes,^29–31^ demonstrating that texture analysis can provide strong holistic cues for quick scene recognition. Previous studies have shown that first and second order (Gray Level Co-occurrence Matrix [GLCM]-based) statistical features are relevant for texture analysis in typical viewing modes.^32–34^ However, it is unclear whether these features are also relevant for the perceived GGS of a mammogram, which is extracted briefly after image onset based on a half-second image presentation. Our hypothesis is that a set of global radiomic features (GRFs), including both first order statistics (FOS) and GLCM-based, can differentiate between mammographic textures that result in strong gist signals (ie, *high-gist*) and those that result in weak ones (ie, *low-gist*). Therefore, this experimental study aims to (i) explore whether a set of GRFs comprising FOS and GLCM-based statistics extracted from mammograms can predict *high-* from *low-gist* images and (ii) identify the most important GRFs that drive the GGS. We focus only on these two extreme sides of the gist spectrum (highest and lowest gist images) to ensure that a clear difference between them at their maximum level is achieved. Moreover, mammographic images’ textural features have been linked to the presence of BC^35^^,^^36^ and elevated BC risk.^37^ Therefore, we included images of normal cancer-free and cancer-containing cases, and high-risk individuals, and considered each class separately to ensure that our model is not capturing the signal related to the presence of BC or elevated BC risk, but rather capturing the gist signal.

In addition to enhancing our comprehension of the gist signal’s nature when dealing with complex textures, the present study could yield insights into radiological errors in screening mammograms. Identifying features on normal images which signal a strong gist of the abnormal (FP) and missed cancer cases which signal a weak gist (false negative [FN]) could help in educating radiologists about possible appearances of FP and FN cases so that their diagnostic errors could be better monitored and drastically reduced. Since BC is the major cause of cancer-related death in females worldwide and the efficiency of mammographic screening interpretation depends largely on the visual expertise of clinician radiologists, errors such as FP and FN are significant.^38^ While FP errors can trigger huge annual healthcare expenses of almost US$3 billion (an example from the United States) and an increased patient anxiety and depression, FN mistakes could result in increased treatment complications and reduced overall survival due to delayed detection of BC.^39^^,^^40^ Hence, providing a better understanding about the underlying reasons for diagnostic errors in the interpretation of screening mammograms is crucially important.

## Materials and methods

Institutional ethics approval of all experiment procedures was obtained from the Human Research Ethics Committee of the University of Sydney (protocol no. 2020/324). Informed consent from all subjects participated in the study was acquired prior to the data collection.

### Participants

Seventeen Australian and New Zealand radiologists and breast physicians were invited to participate in this study. These readers were identified as the “gist expert”, through their participation in the previous gist experiment^15^ where they demonstrated superior ability to detect reliable and accurate gist of the abnormal in mammographic images. Full details of their selection and identification were described in a previous study^15^ which also showed that the superior ability of these “gist experts” was not associated with factors such as their years of experience reading mammograms or number of mammograms interpreted each week. A total of 13 readers (11 radiologists and 2 breast physicians) were recruited.

Prior to the data collection, the 13 participants’ characteristics and general workload details (Table 1) were collected via an online questionnaire. Majority of the participants were female (77%) radiologists (85%) working part-time (69%) as screen readers (92%) for the national screening program BreastScreen Australia and did not undertake a fellowship (62%) but had association with a university or educational institute (54%). At the time of participating in the gist experiment, on average, the participants spent 9.5 h per week reading mammograms and read 237 screening cases per week. With that, 70% of their time was dedicated to reading breast images, while 24.6% of their time was dedicated to reading diagnostic mammograms. The participants also had a median of 17.5 years being a certified BreastScreen reader, 21 years registered as a screen reader, and 21.5 years reading mammograms. Of those who had a fellowship training in mammography reading, the median duration of their training program was 6 months in which they completed it on average of 10 years ago. Lastly, on average, the participants performed 250 breast biopsy examinations in the last 12 months with a median of four times in a month correlating/reviewing radiology-pathology findings for biopsy cases, and three times in a month participating in a Multi-Disciplinary Meeting.

**Table 1.** Participants’ characteristics and general workload details at the time of participating in the gist experiment (*n* = 13).

Characteristics	Count (%)
Gender (female, male)	10 (77%), 3 (23%)
Discipline (radiologist, breast physician)	11 (85%), 2 (15%)
Working full-time (yes, no)	4 (31%), 9 (69%)
Being a screen reader (yes, no)	12 (92%), 1 (8%)
Fellowship-trained in mammography reading (yes, no)	5 (38%), 8 (62%)
Whether affiliated with a university or educational institute (yes, no)	7 (54%), 6 (46%)

	Median	Min-Max	25th-75th percentile
No. of hours per week currently spent in reading mammograms	9.5	1.0-30.0	3.0-19.0
No. of screening cases read per week	237	5-800	150-400
Percentage of time dedicated to reading breast images	70	5-95	45-78
Percentage of time dedicated to reading diagnostic mammograms	24.6	0-90.2	9.0-35.0
No. of years certified as a BreastScreen reader	17.5	0.5-22	7.5-20
No. of years registered as a screen reader	21	2-30	12-25
No. of years reading mammograms	21.5	0-34	8.5-25
Duration of fellowship training in mammography reading (months)	6	6-12	6-12
No. of years since completing fellowship training in mammography reading	10	5-16	0-20.0
No. of breast biopsy examinations performed in the last 12 months	250	30-100	77-400
Frequency of correlating/reviewing radiology-pathology findings for biopsy cases in a month	4	1-100	4-40
Frequency of participating in a Multi-Disciplinary Meeting in a month	3	1-6	3-4

### Mammographic images

**Figure 1.**
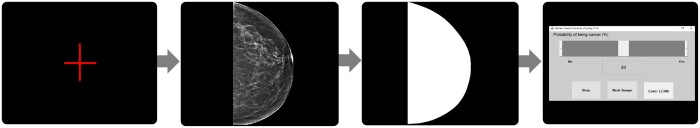
Gist experiment. For a half-second each, a red cross sign was firstly displayed in the centre of the screen, followed by a mammogram and then a corresponding white mask representing the breast area appeared to ensure conclusion of image visual processing. Lastly, without a time limit, a rating screen was shown to collect a gist score (ie, the probability of the image being abnormal) based on a scale of 0-100 (0 means completely confidence that the image was normal while100 means absolute confidence that the image was abnormal).

**Figure 2.**
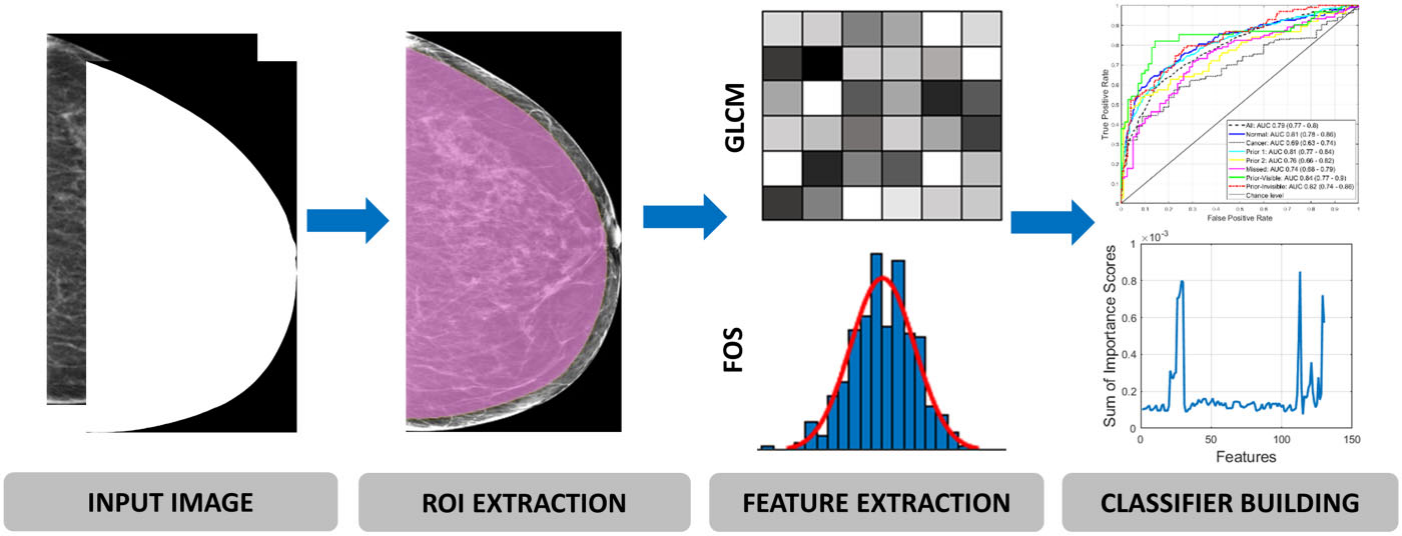
Radiomics analysis workflow. The first step was to obtain input images (unilateral craniocaudal mammograms and masks). Next, a ROI (pink region) was identified using MATLAB’s image erosion algorithm to omit skin-air region. A set of 130 (110 GLCM-based and 20 FOS-based) global radiomic features per image were then computed and finally used to build eight machine learning classification models for predicting *high-gist* from *low-gist* images. Abbreviations: FOS = first order statistics; GLCM = gray level co-occurrence matrix; ROI = region of interest.

For the purpose of conducting this gist experiment and radiomics analysis, mammographic images and binary (black and white) masks were required. A dataset of de-identified 4191 craniocaudal (CC) unilateral digital imaging and communications in medicine (DICOM) mammograms were obtained from the archive of BreastScreen Australia, which were acquired from asymptomatic women aged between 50 and 75. These images were obtained using a variety of vendors, that is, Siemens (Munich, Germany), Hologic (Hologic, Inc., Marlborough, MA, USA), Sectra (Sectra, Linköping, Sweden), Fujifilm (Fujifilm Corporation, Minato City, Tokyo, Japan), Philips (Philips Healthcare, Amsterdam, the Netherlands), and Konica Minolta (Marunouchi, Chiyoda, Tokyo, Japan). Next, binary masks were generated from 4191 DICOM images to extract breast area from its background using a standard thresholding gray level intensity value of 100. Due to computational efficiency reasons (to save computational resources, ie, time and memory), DICOM images and masks were then converted to TIFF format with all the right CC images being flipped to the left for consistent left side chest wall on all images. The TIFF images and binary masks were then cropped based on the maximum breast region size and used for the gist experiment (Fig. 1) and radiomics analysis (Fig. 2).

Since the gist of the abnormal has been reported to be presented in both current and prior images^3–6^^,^^11^^,^^13-16^ and to include various levels of gist strength in our analysis, we included all the eight types of the mammograms in our dataset. The eight types were “Normal” (*n* = 1409), “Cancer” (*n* = 923), “Prior-1” (*n* = 1438), “Prior-2” (*n* = 419), “Prior-3” (*n* = 2), “Missed” (*n* = 375), “Prior-Visible” (*n* = 308), and “Prior-Invisible” (*n* = 755). The “Normal” category included current mammograms of women reported and confirmed to be cancer-free normal by at least two independent expert radiologists (over 20 years of experience) with negative follow up mammograms acquired 2 years after. The “Cancer” had current cancer images contained biopsy proven malignancy. The “Prior-1”, “Prior-2”, and “Prior-3” contained prior no visible cancer signs mammograms, obtained 2, 4, and 6 years before current cancer mammograms, correspondingly. The “Missed”, “Prior-Visible”, and “Prior-Invisible” were categorized by two expert radiologists or a third expert radiologist as an arbitrator if disagreement occurred between the two expert radiologists,^41^ using “Prior-1” category images. These three categories, respectively, included prior mammograms with actional (ie, recall), non-actional (ie, no recall), and no visible cancer signs that were reported as normal, but a later screen showed a biopsy-proven cancer.

### Collecting gist scores

To collect the gist signals/scores from the 13 participants, a previously developed multi-observer gist experimental protocol^4^ was used. Participants were asked to assess the 4191 CC images in 11 batches (381 images per batch selected randomly) and provide the probability of the image being abnormal/gist score based on a scale of 0 to 100 (0 = completely confidence the image was normal while 100 = absolute confidence that the image was abnormal). An extensive description of the data collection process (Fig. 1) was provided in a previous study.^42^ Considering gist has low intra and inter-observer variability, the collected gist scores from the 13 radiologists (who were experts and consistent with each other in their gist scores) were then averaged and used for the analysis, obtaining one gist score per image and eliminating the noise of the gist signal.^11^^,^^14^

### Categorizing high- vs low-gist images

To identify *high-* vs *low-gist* images, the averaged gist score per image was used to classify images into *high-* and *low-gist* based on the 75th and 25th percentiles of the images containing the highest and lowest gist scores, correspondingly. This gave a total of 1048 *high-* and 1048 *low-gist* images (namely “All” image type, including “Prior-3” images which had insufficient number of images, *n* = 2, to form its own group), 162 *high-* and 497 *low-gist* for “Normal”, 545 *high-* and 71 *low-gist* for “Cancer”, 268 *high-* and 370 *low-gist* for “Prior-1”, 73 *high-* and 110 *low-gist* for “Prior-2”, 117 *high-* and 80 *low-gist* for “Missed”, 59 *high-* and 68 *low-gist* for “Prior-Visible”, 92 *high-* and 222 *low-gist* for “Prior-Invisible” image type (Table 2). To accentuate an obvious difference between the highest and lowest gist images, only images in these upper and lower quartiles were used in the analysis.

**Table 2.** Image types included in the study (*n* = 8).

No.	Image type	Description	No. of high-gist images	No. of low-gist images	Total
1	All	Included all the image categories	1048	1048	2096
2	Normal	Current mammograms of women reported and confirmed to be cancer-free normal by at least two independent expert radiologists (20+ years of experience) with negative follow-up mammograms acquired two years after	162	497	659
3	Cancer	Current cancer mammograms of women contained biopsy proven malignancy	545	71	616
4	Prior-1	Prior mammograms with no visible cancer signs acquired two years before current cancer mammograms	268	370	638
5	Prior-2	Prior mammograms with no visible cancer signs acquired four years before current cancer mammograms	73	110	183
6	Missed	Prior-1 mammograms with actionable visible cancer signs (ie, recall) that were reported as normal, but a later screen showed a biopsy-proven cancer	117	80	197
7	Prior-visible	Prior-1 mammograms with non-actionable (ie, not recall) yet visible cancer signs that were reported as normal, but a later screen showed a biopsy-proven cancer	59	68	127
8	Prior-invisible	Prior-1 mammograms with no visible cancer signs that were reported as normal, but a later screen showed a biopsy-proven cancer	92	222	314

### Radiomics and statistical analysis

Using the 4191 images and masks, a set of 130 GRFs per image was extracted using the handcrafted features approach^43^ with our in-house MATLAB-based programs. This is based on the region of interest identified through MATLAB’s binary erosion algorithm using disk-shaped structuring element of 100 radius (distance from the origin to the edge of the disk),^44–46^ to exclude the vendor post-processed background skin-air region in the mammogram in order to obtain the true breast tissue region.^47^ Erosion algorithm was chosen because it is one of most reliable methods of the mathematical morphological image processing which has been widely used for exacting ROI and eliminating the noises in an image.^46^ These features included 110 GLCM-based texture and 20 FOS-based features which have been shown to be valuable descriptors of mammographic appearances in measuring the contrast values of spatial inter-relationships between neighbouring pixels^48–50^ and the distribution of single pixel intensity values within the image region of interest, correspondingly^49^^,^^50^ (Table 3).

**Table 3.** List of global radiomic features (*n* = 130) with feature importance scores.

**No.**^	Feature name	Importance scores from each model							
		All	Normal	Cancer	Missed	Prior-Vis	Prior-Invis	Prior1	Prior2
1	Autocorrelation_1	0.0001	0.0003	0.0003	0.0010	0.0015	0.0005	0.0003	0.0008
2	Autocorrelation_3	0.0001	0.0003	0.0003	0.0009	0.0015	0.0006	0.0003	0.0009
3	Autocorrelation_5	0.0001	0.0003	0.0003	0.0008	0.0013	0.0006	0.0004	0.0009
4	Autocorrelation_9	0.0001	0.0003	0.0003	0.0009	0.0015	0.0005	0.0003	0.0007
5	Autocorrelation_11	0.0001	0.0003	0.0003	0.0008	0.0016	0.0006	0.0004	0.0009
6	Contrast_1	0.0001	0.0003	0.0003	0.0015	0.0007	0.0008	0.0003	0.0018
7	Contrast_3	0.0001	0.0002	0.0003	0.0013	0.0013	0.0005	0.0003	0.0012
8	Contrast_5	0.0001	0.0002	0.0002	0.0009	0.0018	0.0006	0.0003	0.0007
9	Contrast_9	0.0001	0.0002	0.0003	0.0009	0.0019	0.0007	0.0003	0.0008
10	Contrast_11	0.0001	0.0002	0.0003	0.0009	0.0021	0.0006	0.0004	0.0007
11	Correlation_m_1	0.0001	0.0003	0.0002	0.0009	0.0009	0.0007	0.0004	0.0017
12	Correlation_m_3	0.0001	0.0002	0.0003	0.0008	0.0013	0.0006	0.0003	0.0009
13	Correlation_m_5	0.0001	0.0002	0.0003	0.0010	0.0016	0.0005	0.0003	0.0008
14	Correlation_m_9	0.0001	0.0002	0.0002	0.0009	0.0016	0.0005	0.0003	0.0010
15	Correlation_m_11	0.0001	0.0002	0.0003	0.0013	0.0020	0.0006	0.0004	0.0007
16	Correlation_p_1	0.0001	0.0003	0.0003	0.0010	0.0008	0.0007	0.0004	0.0019
17	Correlation_p_3	0.0001	0.0002	0.0003	0.0007	0.0013	0.0006	0.0003	0.0009
18	Correlation_p_5	0.0001	0.0002	0.0003	0.0010	0.0014	0.0005	0.0003	0.0008
19	Correlation_p_9	0.0001	0.0002	0.0002	0.0009	0.0016	0.0005	0.0004	0.0010
20	Correlation_p_11	0.0001	0.0002	0.0003	0.0013	0.0020	0.0006	0.0004	0.0007
21	Cluster_prominence_1	0.0003	0.0011	0.0006	0.0051	0.0161	0.0027	0.0016	0.0043
22	Cluster_prominence_3	0.0003	0.0013	0.0006	0.0050	0.0136	0.0028	0.0015	0.0040
23	Cluster_prominence_5	0.0003	0.0013	0.0005	0.0055	0.0124	0.0026	0.0017	0.0048
24	Cluster_prominence_9	0.0003	0.0012	0.0005	0.0055	0.0139	0.0035	0.0018	0.0052
25	Cluster_prominence_11	0.0003	0.0013	0.0006	0.0050	0.0121	0.0029	0.0017	0.0044
26	Cluster_shade_1	0.0008*	0.0030*	0.0009*	0.0055	0.0249*	0.0071*	0.0035*	0.0119*
27	Cluster_shade_3	0.0008*	0.0033*	0.0010*	0.0051	0.0262*	0.0068*	0.0035*	0.0126*
28	Cluster_shade_5	0.0008*	0.0034*	0.0009*	0.0051	0.0277*	0.0075*	0.0036*	0.0138*
29	Cluster_shade_9	0.0009*	0.0038*	0.0009*	0.0066	0.0303*	0.0088*	0.0040*	0.0148*
30	Cluster_shade_11	0.0009*	0.0040*	0.0009*	0.0059	0.0266*	0.0092*	0.0039*	0.0119*
31	Dissimilarity_1	0.0001	0.0003	0.0003	0.0011	0.0008	0.0007	0.0003	0.0018
32	Dissimilarity_3	0.0001	0.0003	0.0002	0.0010	0.0008	0.0005	0.0003	0.0013
33	Dissimilarity_5	0.0001	0.0003	0.0002	0.0011	0.0015	0.0005	0.0003	0.0011
34	Dissimilarity_9	0.0001	0.0002	0.0003	0.0012	0.0011	0.0007	0.0003	0.0011
35	Dissimilarity_11	0.0001	0.0002	0.0003	0.0011	0.0012	0.0007	0.0004	0.0007
36	Energy_1	0.0001	0.0003	0.0002	0.0013	0.0012	0.0008	0.0004	0.0013
37	Energy_3	0.0001	0.0004	0.0003	0.0013	0.0011	0.0008	0.0004	0.0016
38	Energy_5	0.0001	0.0004	0.0003	0.0011	0.0013	0.0007	0.0003	0.0015
39	Energy_9	0.0001	0.0006	0.0003	0.0009	0.0019	0.0007	0.0003	0.0012
40	Energy_11	0.0001	0.0004	0.0002	0.0013	0.0016	0.0007	0.0004	0.0016
41	Entropy_1	0.0002	0.0003	0.0004	0.0012	0.0024	0.0010	0.0006	0.0012
42	Entropy_3	0.0002	0.0003	0.0004	0.0015	0.0017	0.0007	0.0004	0.0013
43	Entropy_5	0.0001	0.0003	0.0003	0.0015	0.0022	0.0008	0.0004	0.0011
44	Entropy_9	0.0002	0.0004	0.0004	0.0013	0.0018	0.0007	0.0004	0.0012
45	Entropy_11	0.0002	0.0004	0.0003	0.0015	0.0025	0.0008	0.0004	0.0017
46	Homogeneity_m_1	0.0002	0.0003	0.0004	0.0009	0.0021	0.0009	0.0004	0.0016
47	Homogeneity_m_3	0.0001	0.0003	0.0004	0.0013	0.0017	0.0006	0.0003	0.0011
48	Homogeneity_m_5	0.0001	0.0003	0.0003	0.0019	0.0019	0.0006	0.0004	0.0010
49	Homogeneity_m_9	0.0001	0.0003	0.0004	0.0017	0.0013	0.0006	0.0004	0.0015
50	Homogeneity_m_11	0.0001	0.0003	0.0003	0.0023	0.0013	0.0007	0.0004	0.0012
51	Homogeneity_1	0.0002	0.0003	0.0003	0.0008	0.0021	0.0009	0.0004	0.0019
52	Homogeneity_3	0.0001	0.0003	0.0004	0.0016	0.0018	0.0007	0.0003	0.0012
53	Homogeneity_5	0.0001	0.0003	0.0003	0.0018	0.0020	0.0007	0.0004	0.0010
54	Homogeneity_9	0.0002	0.0003	0.0004	0.0014	0.0018	0.0006	0.0004	0.0012
55	Homogeneity_11	0.0001	0.0003	0.0003	0.0025	0.0018	0.0006	0.0004	0.0011
56	Maximum_probability_1	0.0001	0.0003	0.0003	0.0013	0.0014	0.0010	0.0004	0.0013
57	Maximum_probability_3	0.0001	0.0004	0.0003	0.0013	0.0013	0.0008	0.0003	0.0015
58	Maximum_probability_5	0.0001	0.0004	0.0002	0.0011	0.0013	0.0008	0.0004	0.0014
59	Maximum_probability_9	0.0001	0.0005	0.0003	0.0011	0.0017	0.0007	0.0003	0.0012
60	Maximum_probability_11	0.0001	0.0004	0.0002	0.0012	0.0018	0.0007	0.0004	0.0014
61	Sum_of_sqaures_variance_1	0.0001	0.0003	0.0003	0.0009	0.0013	0.0005	0.0003	0.0009
62	Sum_of_sqaures_variance_3	0.0001	0.0003	0.0003	0.0009	0.0016	0.0005	0.0003	0.0010
63	Sum_of_sqaures_variance_5	0.0001	0.0003	0.0003	0.0009	0.0014	0.0005	0.0003	0.0009
64	Sum_of_sqaures_variance_9	0.0001	0.0003	0.0003	0.0009	0.0017	0.0004	0.0003	0.0009
65	Sum_of_sqaures_variance_11	0.0001	0.0003	0.0003	0.0010	0.0015	0.0004	0.0003	0.0009
66	Sum_average_1	0.0001	0.0004	0.0003	0.0011	0.0012	0.0006	0.0003	0.0015
67	Sum_average_3	0.0001	0.0004	0.0004	0.0010	0.0013	0.0007	0.0004	0.0014
68	Sum_average_5	0.0001	0.0004	0.0004	0.0010	0.0014	0.0006	0.0004	0.0013
69	Sum_average_9	0.0001	0.0004	0.0004	0.0009	0.0013	0.0006	0.0004	0.0012
70	Sum_average_11	0.0001	0.0004	0.0003	0.0010	0.0017	0.0006	0.0003	0.0014
71	Sum_variance_1	0.0001	0.0003	0.0003	0.0009	0.0017	0.0006	0.0003	0.0010
72	Sum_variance_3	0.0001	0.0003	0.0003	0.0010	0.0015	0.0005	0.0003	0.0011
73	Sum_variance_5	0.0001	0.0003	0.0003	0.0011	0.0015	0.0006	0.0003	0.0011
74	Sum_variance_9	0.0001	0.0003	0.0003	0.0010	0.0017	0.0005	0.0004	0.0011
75	Sum_variance_11	0.0001	0.0003	0.0003	0.0011	0.0018	0.0006	0.0003	0.0012
76	Sum_entropy_1	0.0001	0.0006	0.0003	0.0015	0.0020	0.0009	0.0004	0.0017
77	Sum_entropy_3	0.0001	0.0006	0.0004	0.0015	0.0014	0.0007	0.0004	0.0019
78	Sum_entropy_5	0.0001	0.0006	0.0003	0.0017	0.0013	0.0010	0.0004	0.0014
79	Sum_entropy_9	0.0001	0.0006	0.0003	0.0018	0.0020	0.0007	0.0004	0.0015
80	Sum_entropy_11	0.0001	0.0006	0.0003	0.0017	0.0019	0.0008	0.0005	0.0020
81	Difference_variance_1	0.0001	0.0003	0.0003	0.0015	0.0008	0.0008	0.0003	0.0017
82	Difference_variance_3	0.0001	0.0002	0.0003	0.0015	0.0011	0.0005	0.0003	0.0011
83	Difference_variance_5	0.0001	0.0002	0.0002	0.0009	0.0018	0.0006	0.0004	0.0007
84	Difference_variance_9	0.0001	0.0002	0.0002	0.0010	0.0019	0.0006	0.0003	0.0009
85	Difference_variance_11	0.0001	0.0002	0.0003	0.0009	0.0021	0.0006	0.0004	0.0008
86	Difference_entropy _1	0.0001	0.0003	0.0003	0.0012	0.0013	0.0008	0.0004	0.0017
87	Difference_entropy _3	0.0001	0.0003	0.0003	0.0010	0.0010	0.0007	0.0003	0.0017
88	Difference_entropy _5	0.0001	0.0003	0.0002	0.0012	0.0012	0.0006	0.0004	0.0010
89	Difference_entropy _9	0.0001	0.0002	0.0003	0.0015	0.0011	0.0008	0.0004	0.0013
90	Difference_entropy _11	0.0001	0.0002	0.0003	0.0017	0.0011	0.0008	0.0005	0.0008
91	Information_measure_of_correlation1_1	0.0001	0.0003	0.0003	0.0011	0.0016	0.0010	0.0004	0.0028
92	Information_measure_of_correlation1_3	0.0001	0.0003	0.0003	0.0008	0.0018	0.0008	0.0003	0.0014
93	Information_measure_of_correlation1_5	0.0001	0.0003	0.0003	0.0011	0.0014	0.0007	0.0004	0.0011
94	Information_measure_of_correlation1_9	0.0001	0.0002	0.0003	0.0011	0.0009	0.0009	0.0004	0.0016
95	Information_measure_of_correlation1_11	0.0001	0.0003	0.0004	0.0017	0.0009	0.0007	0.0004	0.0008
96	Information_measure_of_correlation2_1	0.0001	0.0004	0.0003	0.0013	0.0017	0.0009	0.0004	0.0019
97	Information_measure_of_correlation2_3	0.0001	0.0004	0.0003	0.0011	0.0020	0.0008	0.0004	0.0015
98	Information_measure_of_correlation2_5	0.0001	0.0003	0.0003	0.0013	0.0017	0.0006	0.0004	0.0016
99	Information_measure_of_correlation2_9	0.0001	0.0002	0.0003	0.0012	0.0011	0.0009	0.0004	0.0017
100	Information_measure_of_correlation2_11	0.0001	0.0002	0.0003	0.0017	0.0014	0.0005	0.0004	0.0010
101	Inverse_difference_normalized _1	0.0001	0.0003	0.0003	0.0011	0.0009	0.0007	0.0004	0.0019
102	Inverse_difference_normalized _3	0.0001	0.0003	0.0002	0.0011	0.0011	0.0006	0.0003	0.0014
103	Inverse_difference_normalized _5	0.0001	0.0003	0.0003	0.0011	0.0016	0.0004	0.0003	0.0011
104	Inverse_difference_normalized _9	0.0001	0.0002	0.0003	0.0012	0.0015	0.0007	0.0004	0.0012
105	Inverse_difference_normalized _11	0.0001	0.0003	0.0003	0.0013	0.0011	0.0006	0.0004	0.0007
106	Inverse_difference_moment_normalized _1	0.0001	0.0004	0.0003	0.0014	0.0008	0.0007	0.0003	0.0020
107	Inverse_difference_moment_normalized _3	0.0001	0.0002	0.0003	0.0013	0.0013	0.0005	0.0003	0.0011
108	Inverse_difference_moment_normalized _5	0.0001	0.0002	0.0002	0.0009	0.0015	0.0005	0.0003	0.0007
109	Inverse_difference_moment_normalized _9	0.0001	0.0002	0.0003	0.0011	0.0019	0.0006	0.0003	0.0009
110	Inverse_difference_moment_normalized _11	0.0001	0.0002	0.0003	0.0010	0.0019	0.0006	0.0004	0.0008
111	Mean	0.0002	0.0008	0.0004	0.0013	0.0019	0.0010	0.0005	0.0018
112	Standard_deviation	0.0005	0.0029*	0.0006	0.0085*	0.0262*	0.0050*	0.0025	0.0069
113	Skewness	0.0009*	0.0014	0.0006	0.0056	0.0114	0.0047*	0.0024	0.0065
114	Kurtosis	0.0002	0.0004	0.0008*	0.0019	0.0017	0.0014	0.0006	0.0012
115	Minimum	0.0001	0.0001	0.0003	0.0005	0.0004	0.0002	0.0001	0.0004
116	5th_percentile	0.0002	0.0007	0.0006	0.0016	0.0025	0.0009	0.0005	0.0009
117	10th_percentile	0.0002	0.0008	0.0004	0.0016	0.0015	0.0015	0.0006	0.0014
118	15th_percentile	0.0002	0.0007	0.0004	0.0021	0.0020	0.0021	0.0006	0.0017
119	20th_percentile	0.0002	0.0009	0.0004	0.0017	0.0026	0.0023	0.0007	0.0018
120	25th_percentile	0.0003	0.0011	0.0004	0.0017	0.0032	0.0020	0.0007	0.0024
121	Median	0.0004	0.0018	0.0003	0.0022	0.0027	0.0019	0.0010	0.0026
122	75th_percentile	0.0002	0.0008	0.0003	0.0018	0.0013	0.0015	0.0006	0.0011
123	80th_percentile	0.0002	0.0005	0.0003	0.0015	0.0016	0.0011	0.0004	0.0010
124	85th_percentile	0.0001	0.0004	0.0004	0.0016	0.0018	0.0010	0.0005	0.0013
125	90^th^_percentile	0.0001	0.0006	0.0004	0.0024	0.0036	0.0013	0.0006	0.0015
126	95th_percentile	0.0003	0.0017	0.0005	0.0048	0.0094	0.0026	0.0012	0.0032
127	Maximum	0.0002	0.0003	0.0003	0.0013	0.0031	0.0007	0.0006	0.0010
128	Range_all (maximum less minimum)	0.0002	0.0004	0.0003	0.0017	0.0032	0.0011	0.0006	0.0021
129	Range5 (95^th^ percentile less 5th percentile)	0.0008*	0.0017	0.0011*	0.0093*	0.0318*	0.0053*	0.0035*	0.0107*
130	Range2 (99th percentile less 1st percentile)	0.0006	0.0033*	0.0008*	0.0087*	0.0165	0.0060*	0.0025	0.0082

Feature nos. 1-110 are GLCM-based features^49–51^ with feature parameters as 1, 3, 5, 9, and 11 being pixel distance between the pixel of interest and its neighbour. Feature nos. 111-130 are FOS-based features.^50^^,^^51^

* and highlighted in grey = important features based on their importance scores from the machine learning model. The larger the importance scores, the more important the features were for the model.

Abbreviations: FOS = first order statistics; GLCM = gray level co-occurrence matrix.

To predict *high-* from *low-gist* images, the 130 GRFs, derived from the corresponding image types (“All”, “Normal”, “Cancer”, “Prior-1”, “Prior-2”, “Missed”, “Prior-Visible”, and “Prior-Invisible”—Table 2), were then used to build eight separate machine learning (ML) random forest classifiers (*All*, *Normal*, *Cancer*, *Prior-1*, *Prior-2*, *Missed*, *Prior-Visible*, and *Prior-Invisible*) based on the ensemble of 500 decision trees using bootstrap aggregation method.^51^^,^^52^ The random forest with bootstrap aggregation technique was chosen because of its built-in feature selection function that can minimize feature-overfitting and imbalanced data issues (ie, oversampling unique observations from the minority class to balance the data), and automatic estimation of feature importance for generating interpretable classifier.^53^^,^^54^ Useful features for each predictive model were also recognized through feature important analysis of their importance scores estimated using MATLAB’s predictor importance algorithm. To determine the useful GRFs based their total importance scores from each model, a scree test of exploratory factor analysis^55^ was utilized. Importance scores show the usefulness of each feature in constructing the decision trees within the model, with larger values mean more impact on the predictions made by the model.

In evaluating the performance of the models, we trained and validated each model using the 10-fold cross-validation approach,^53^^,^^56^ a reliable, unbiased, and accurate validation method for estimating the model’s generalization performance. This involved randomly splitting the dataset into 10 groups, and while the first group was used once to test the predictive performance of the model, the rest of the groups were used to train the model. This process was repeated 10 times until each group was used once as a test set. The area under the receiver operating characteristic curve (AUC) was then used to evaluate the overall performance of the models for distinguishing *high-* from *low-gist* images.

MATLAB R2022a (MathWorks, Natick, MA, USA) was used to complete all radiomics and statistical analysis.

## Results

### GRFs predicting high- vs low-gist images

**Figure 3.**
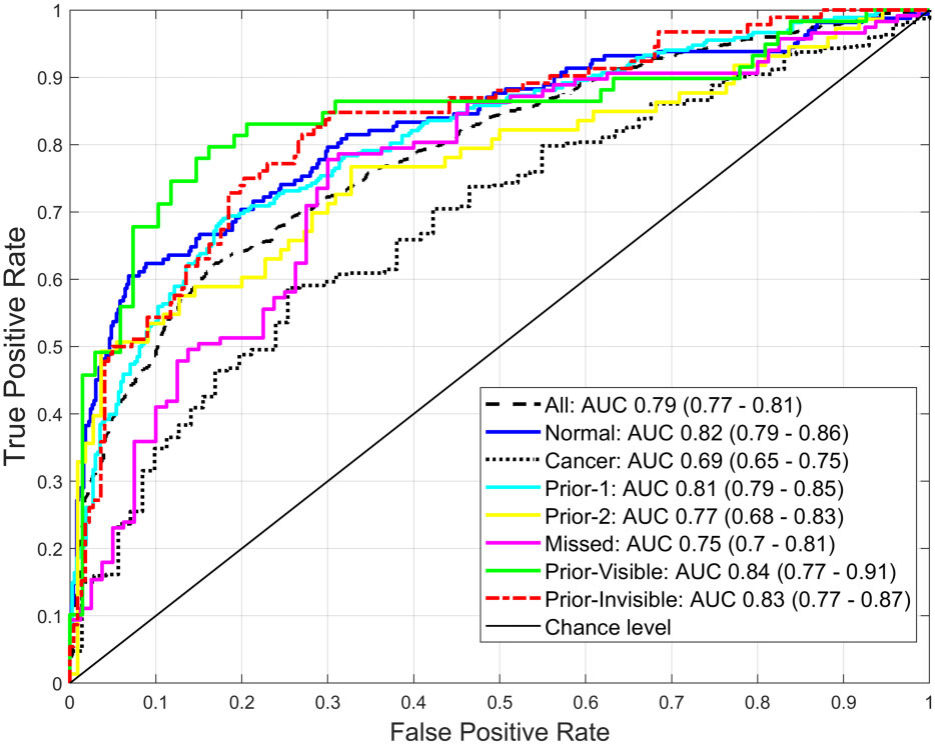
Area under the receiver operating characteristic curve (AUC) for eight classifiers. When differentiating *high-* from *low-gist* images, all eight models using global radiomic features from each image category performed relatively well with the *Prior-Visible* images category model achieved the highest AUC of 0.84 (95% CI, 0.77-0.91) while the *Cancer* model had an AUC of 0.69 (95% CI, 0.65-0.75).

The overall performance of the eight models (*All*, *Normal*, *Cancer*, *Prior-1*, *Prior-2*, *Missed*, *Prior-Visible*, *Prior-Invisible*) is shown in Fig. 3. When differentiating *high-* from *low-gist* images, the *Prior-Visible* model reached the highest AUC of 0.84 (95% CI, 0.77-0.91) followed by the *Prior-Invisible* (0.83, 0.77-0.87), *Normal* (0.82, 0.79-0.86), *Prior-1* (0.81, 0.79-0.85), *All* (0.79, 0.77-0.81), *Prior-2* (0.77, 0.68-0.83), *Missed* (0.75, 0.70-0.81), and *Cancer* model (0.69, 0.65-0.75).

### Important GRFs

**Figure 4.**
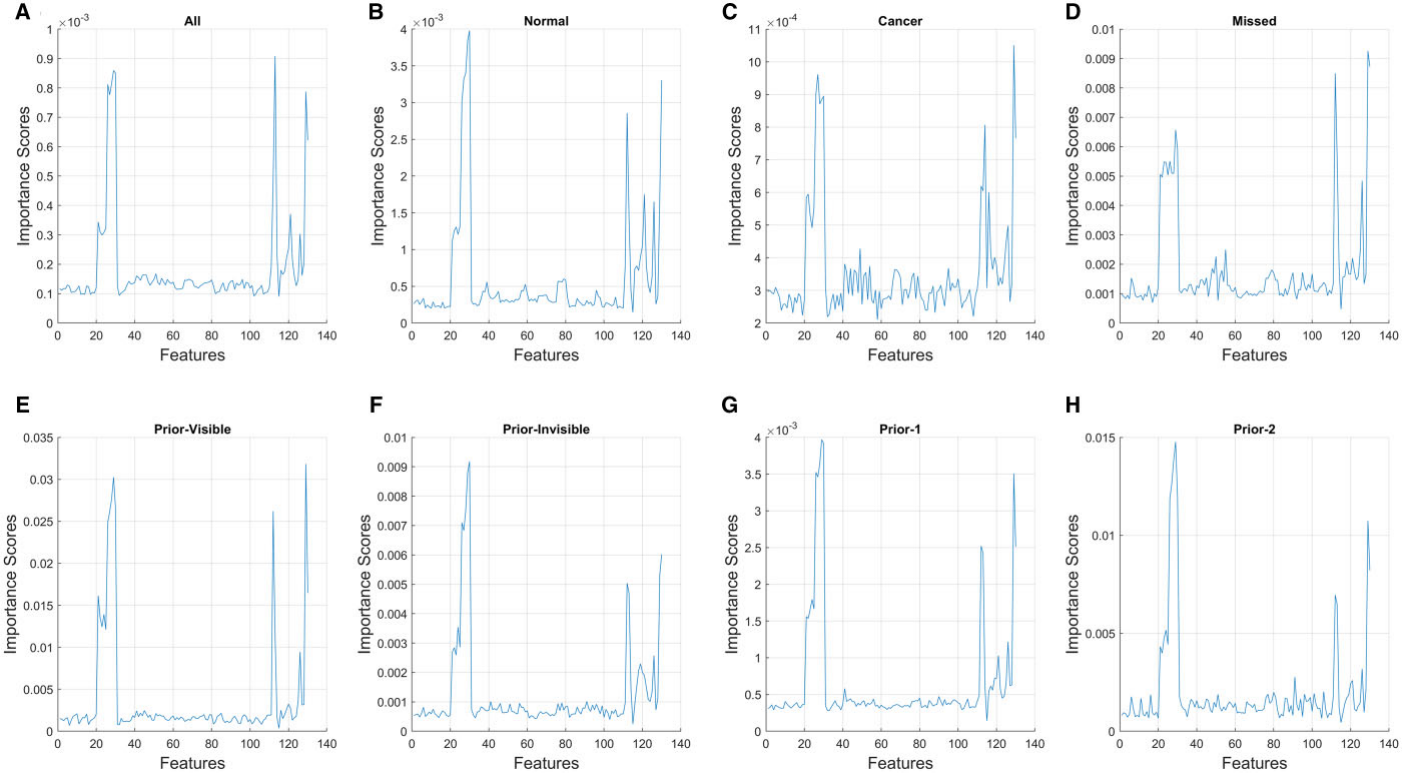
Line plot showing feature importance scores of the 130 features (no. 1-130) in each classifier (A)-(H). As shown, pattern of important features appears to be similar across the eight classifiers.

**Figure 5.**
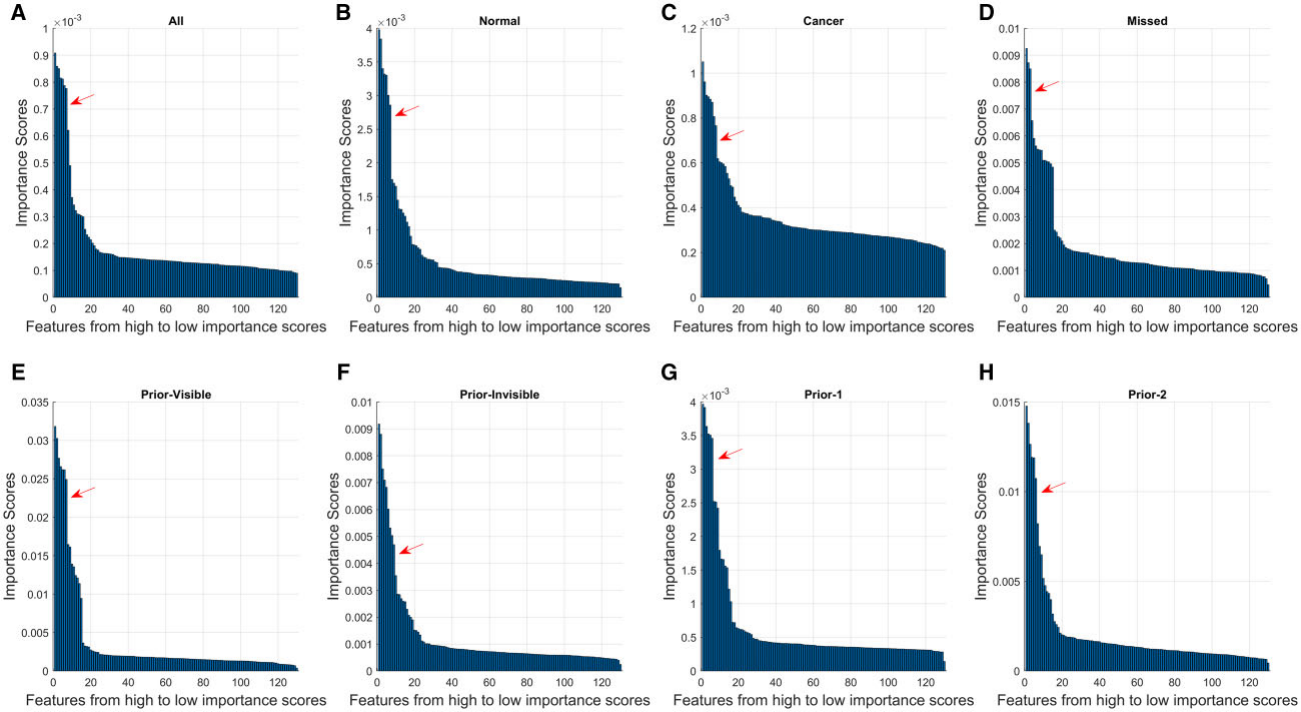
Scree bar chart for each of the eight classifiers showing important features (ranked from high to low importance scores) determined based on the identified steep slope (as shown in red arrow). Seven, seven, eight, three, seven, nine, six, and six most important features were identified for *All* (A), *Normal* (B), *Cancer* (C), *Missed* (D), *Prior-Visible* (E), *Prior-Invisible* (F), *Prior-1* (G), *Prior-2* (H) model, respectively.

Table 3 and Fig. 4 show the feature importance scores of the 130 GRFs derived from each of the eight models. When comparing each feature’s importance score, ranked from high to low, in each individual model (Fig. 5), five features appear to be significant for most of the models. These features were *cluster shade* (feature no. 26-30, calculated across five orientations) of the GLCM,^48–50^*standard deviation* (feature no. 112), *skewness* (feature no. 113), *kurtosis* (feature no. 114), and *range* (feature no. 129 and 130, calculated across two dimensions) of the FOS.^49^^,^^50^*Cluster shade* is a measure of the symmetry distribution in an image. *Standard deviation* describes the variation or spreading of gray level intensity from the mean value. *Skewness* is a measure of the unevenness of the distribution. *Kurtosis* measures the extreme values of the distribution in the image. Also, *range* describes the difference between 95th and 5th percentile (feature no. 129), and 99th and 1st percentile of image gray level values (feature no. 130).

## Discussion

For almost 50 years, research has shown that the holistic rapid visual impression of an image (ie, GGS) can convey a great amount of useful information about an image abnormality in the medical image interpretation and diagnostic process, aiming to significantly reduce FN and FP errors.^2^^,^^18^^,^^26^ Recent medical image perception studies have recognized the existence of GGS shown to be valuable for providing information about the presence of BC to facilitate the improvement of early BC detection and future risk prediction.^3–6^^,^^13-16^ However, little is known about which global computer extracted image features are correlated to the intensity of the GGS.^21^ Here, for the first time, using radiomics approach,^43^ we built upon the previous works and evaluated GRFs that drive the strength of the gist signals. We hypothesized that GRFs of *high-gist* mammograms are different from *low-gist* ones, based on the efficacy of radiomics in mammography^43^ and the GGS, independent of lesion characteristics (eg, breast density), in identifying image abnormality.^3–5^^,^^11^^,^^13^^,^^14^^,^^16^^,^^24^ We also examined which features are more important for distinguishing between *high-gist* mammograms from *low-gist* ones.

The results obtained from our eight ML classification models (*All*, *Normal*, *Cancer*, *Prior-1*, *Prior-2*, *Missed*, *Prior-Visible*, *Prior-Invisible*) demonstrated that the GRFs had the ability to accurately differentiate *high-* from *low-gist* images with as high as an AUC of 0.84 (Fig. 3). Interestingly, although the GGS has been shown to be helpful for predicting cancer images,^4^^,^^13^ the *Cancer* model in our study achieved smaller AUC value of 0.69 in discriminating *high-* from *low-gist* cancer mammograms when compared to other seven models (AUCs of 0.75-0.84). This could be because of the large imbalanced dataset (*n* = 474) between the *high-gist* (*n* = 545) and *low-gist* (*n* = 71) images in the “Cancer” image category, making it difficult for the *Cancer* model to optimally learn the difference, thereby limiting its predictive capability. Nevertheless, our overall promising findings (ie, up to 0.84 AUC) indicate the significance of the GRFs in predicting *high-gist* images which could potentially be used to guide the proper use of the GGS to support BC detection and prediction process.

Moreover, if the GGS can be considered as a BC risk factor, similar to breast density or genetic risk factor, our work may be applied as a supplemented artificial vision intelligence tool to signal clinician about an elevated risk of a positive cancer finding in mammographic screening.^3^^,^^5^^,^^13^ It might also be used to augment the recent deep learning model^57^ to further advance the accuracy of BC detection in screening mammography especially since the GGS has been shown to be helpful in improving the performance of the deep learning model in detecting BC.^14^ This could not only assist in lessening the possibility of FP and FN errors but also optimizing personalized screening strategy (eg, shorter screening interval) and increasing the chance of BC being detected early with improved therapy outcomes and survivorship. Conversely, more research efforts are needed to further evaluate and validate its clinical usefulness.

Interestingly, although studies^3^^,^^58^ indicated that GGS of future cancerous abnormalities can be detected in fine details of the parenchymal texture of high-pass filtered mammograms, other works revealed that radiologists extract the GGS using low-resolution peripheral vision prior to detailed foveal processing of any image areas when separating abnormal from normal mammograms.^1^^,^^10^^,^^24^ Our findings supported the relevance of the FOS and GLCM-based second-order textural features to the GGS, extracted by radiologists’ peripheral vision. Prior studies^3^^,^^13^ showed that observers failed to localize BC using such gist signal, indicating that the gist signal in the context of medical images is concordant with findings from basic vision sciences where observers struggle with tasks involving distinguishing phase differences^59^ and show uncertainty in localizing bisection.^60^ To move beyond qualitative evidence, a specific proposal for peripheral “texture processing” and its relevance to mammography are required. The current best theory for a model of capturing the appearance of textures is the Texture Tiling Model^61^^,^^62^ which implicates the visual system with the summary statistics computed using the second-order statistics of primary visual cortex over local pooling regions.

In the context of basic vision science, the importance of texture features from non-medical images in identifying the global gist of natural scenes was discovered.^29–31^ Our work based on breast medical images also showed similar results, in that, five useful features involving one GLCM-based textural and four FOS-based features (Table 3) from the ML models were found. In concordance with the suggestions from basic vision science studies,^33^^,^^34^^,^^63^ features that are related to noticeable differences in the first order luminance, that is, *range* and *standard_deviation*, were among important features in the classification tasks. In particular, *range5* (feature no. 129) seems to be valuable in all the model except *Normal*, signifying that a high GGS in most image types may be motivated by the overall low range (difference between the 95th and 5th percentiles) of the image gray level value, resembling cancer characteristics. Similarly, among the second-order features, *cluster shade* (feature no. 26-30) exhibited importance in all tasks except for one, suggesting that the “uniformity of mammographic texture” could contribute to the radiologists’ initial impression about the abnormality of a case. In this case, these features may be used as an indicator of whether a strong gist of the abnormal is present in an image, which could contribute to the early detection and risk prediction of BC. However, larger research is required to further examine the effectiveness of these features.

There were a few limitations in our study. First, this was the first exploratory study investigating the capability of handcrafted GRFs (ie, 110 GLCM-based texture and 20 FOS-based features) derived from 130 CC unilateral view mammograms^64^ in predicting *high-gist* from *low-gist* images based on the gist scores of thirteen “gist experts”.^15^ The mediolateral oblique (MLO) view was not used in this study. While the entire images were viewed by the readers in the gist experiment, the background skin-air region of the images had to be excluded using erosion algorithm, because it has been enhanced by the vendors, to obtain the true breast region for GRF extraction. The results may be only applicable to the images and gist experts with characteristic/background (eg, being a screen reader with high screening workload) similar to the sample used in the study. Secondly, useful GRFs were determined based on their importance scores using a scree test^55^ (Fig. 4), which may be less explicit for some models, for instance, the *Missed* model when compared to the *Normal* model. Also, only “Prior-1” images (attained two years prior to current cancer mammograms) were used to categorize “Missed”, “Prior-Visible”, and “Prior-Invisible” images. As with all model building, the output of the model is constrained by the parameters entered into the model. So, forthcoming studies should examine other prior mammograms acquired beyond 2 years before, MLO, raw^47^ and high-pass filtered bilateral mammograms,^58^ digital breast tomosynthesis images,^65^ and images from other vendors (eg, GE, GE Healthcare, Chicago, IL, USA). These images may also contain valuable information to suggest an abnormality and/or an increased future malignancy risk, and therefore may result in different gist scores and GRFs. Also, gist experts from other location and using other morphological image processing methods (such as corrosion and closing^66^) may produce different results. Lastly, other potential important GRFs (eg, *cluster prominence*, feature no. 21-25) and types of GRFs (eg, deep learning features^11^) should also be analysed.

## Conclusions

To summarize, this study suggests that a set of quantitative GRFs derived from mammographic images can accurately predict *high-* from *low-gist* images with five useful GRFs (one GLCM-based texture and four FOS-based features) discovered and emphasized. These findings are critically important in providing better understanding of the mammographic image characteristics that drive the strength of the GGS, which could be exploited to advance BC screening and risk prediction, enabling early detection and treatment of BC thereby further reducing BC-related deaths.

## Data Availability

The dataset that supports the findings of this study is not publicly available as it contains proprietary information that the authors acquired through a license. Data are, however, available from the authors upon reasonable request.
